# E2F1 combined with *LINC01004* super-enhancer to promote hepatocellular carcinoma cell proliferation and metastasis

**DOI:** 10.1186/s13148-023-01428-6

**Published:** 2023-01-31

**Authors:** Jingxuan Li, Jiying Wang, Yanping Wang, Xueyan Zhao, Tao Su

**Affiliations:** 1 Shandong Provincial Key Laboratory of Animal Disease Control and Breeding, Institute of Animal Science and Veterinary Medicine, Shandong Academy of Agricultural Sciences, Jinan, China; 2 Medical Integration and Practice Center, Cheeloo College of Medicine, Shandong University, Jinan, China

**Keywords:** lncRNA, Super-enhancer, Hepatocellular carcinoma, E2F1

## Abstract

**Introduction:**

Super-enhancer-associated lncRNAs play important roles in the occurrence and development of malignant tumors, including hepatocellular carcinoma (HCC).

**Objectives:**

The current work aimed to identify and characterize super-enhancer-associated lncRNAs in the pathogenesis of HCC.

**Methods:**

H3K27ac ChIP-seq data from HepG2 cell line and two HCC tissues were used to identify super-enhancer-associated lncRNAs in HCC. JQ-1 treatment and CRISPR-dCas9 system were performed to confirm super-enhancer activity. Quantitative real-time PCR (qPCR), ChIP-qPCR, and dual-luciferase reporter system assay demonstrated the regulation of E2F1 on super-enhancer. Functional loss experiment was used to identify the function of *LINC01004*.

**Results:**

In this study, we identified and characterized *LINC01004*, a novel super-enhancer-associated lncRNA, as a crucial oncogene in HCC. *LINC01004* was upregulated in liver cancer tissues and was associated with poor patient prognosis. Moreover, *LINC01004* promoted cell proliferation and metastasis of HCC. The binding of E2F1 to the super-enhancer could promote the transcription of *LINC01004*, while the inhibition of super-enhancer activity decreased *LINC01004* expression.

**Conclusion:**

This finding might provide mechanistic insights into the molecular mechanisms underlying hepatocarcinogenesis and the biological function of super-enhancer. *LINC01004* can serve as a potential therapeutic target for HCC patient.

**Graphical abstract:**

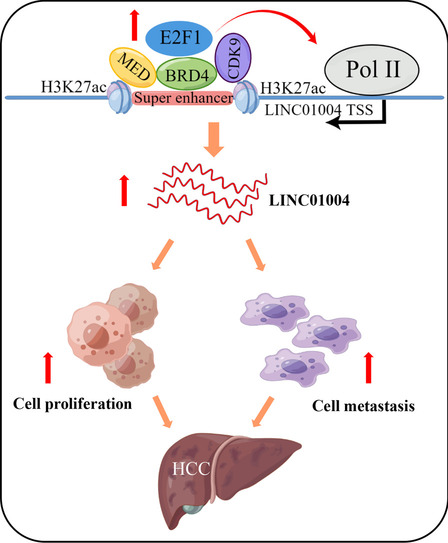

**Supplementary Information:**

The online version contains supplementary material available at 10.1186/s13148-023-01428-6.

## Introduction

Primary liver cancer is the sixth most common malignancy, which also ranks the third most common cause of cancer-related death [1]. Hepatocellular carcinoma (HCC) is the main histological subtype of primary liver cancer (comprising 75–85% of cases) [1]. HCC is also a devastating disease with limited therapeutic approaches and relatively low 5-year survival rate [2]. Although recent diagnosis of HCC has been greatly improved, a large portion of patients are still diagnosed in advanced stage [3]. Therefore, an urgent need exists to understand the pathogenesis of HCC, detail the illustration of genetic landscape during hepatocarcinogenesis, and discover the novel tumor biomarkers.

Abnormal transcription of oncogenes has been proved to be an important reason for hepatocarcinogenesis [4]. Recent advance indicates that some oncogenes transcription is frequently driven by enhancer which is an active transcription regulatory element [5, 6]. Super-enhancer (SE) is an exceptionally large cluster of enhancers that synergistically drives gene transcriptions [7]. Super-enhancers have high levels of histone-3-lysine-27 acetylation (H3K27Ac) and histone-3-lysine-4 methylation modification, and was densely occupied by various transcription factors [7, 8]. Emerging evidence indicates that cancer cells use SEs to drive transcription of oncogenes, ranging from protein coding genes to non-coding RNAs [9–11].

Long non-coding RNAs (lncRNAs) are transcripts over 200 nucleotides, which are involved in many key biological processes including malignancy [12–14]. Many tumor-associated lncRNAs are also regulated by super-enhancers [15–19]. In HCC, HCCL5 was found to be a novel super-enhancer-associated lncRNA, which was upregulated during epithelial to mesenchymal transition (EMT) and facilitated the EMT process. Simultaneously, the binding of ZEB1 to the SE promoted the expression of HCCL5 [20]. LncRNA-DAW was another super-enhancer-associated lncRNA in HCC. LncRNA-DAW was driven by a liver-specific SE and promoted tumor growth. Mechanistically, lncRNA-DAW antagonized the suppressive effect of EZH2 on Wnt2 expression, leading to the activation of the Wnt/β-catenin pathway [21]. It will be of value to identify and characterize such super-enhancer-associated lncRNAs in the pathogenesis of HCC.

In this study, we used H3K27ac ChIP-seq data from HCC cells and tissues to identify SEs, and discovered 25 super-enhancer-associated lncRNAs. *LINC01004* was identified as one of the novel lncRNAs driven by SE. *LINC01004* was frequently upregulated in liver cancer tissues and was associated with poor patient prognosis. The binding of E2F1 to the SE could promote the transcription of *LINC01004*. However, the inhibition of SE activity could decrease *LINC01004* expression. Functionally, knockdown of *LINC01004* inhibited HCC cell proliferation and metastasis. Together, this study characterized *LINC01004* as a novel super-enhancer-driven lncRNA regulated by E2F1 promoting HCC cell proliferation and metastasis. Our data also suggest that *LINC01004* may serve as a novel prognostic biomarker and therapeutic target in HCC.

## Materials and methods

### Experimental animals and ethic

Mice used in this research were 5-week-old male nude mice. The mice were kept in SPF standard animal house with plenty of food and water. The xenograft model was established by subcutaneous injection of tumor cells. The methods were carried out in accordance with the approved guidelines. This study was approved by Ethics Committee of Shandong University School of Clinical Medicine (SDULCLL2022-2-2).

### Identification of super-enhancers and associated lncRNAs

The H3K27ac ChIP-seq data of hepatocellular carcinoma were obtained from ENCODE project (ENCSR000AMO) and GEO database (GSE112221). Super-enhancers (SEs) were identified by ROSE (https://bitbucket.org/youngcomputation/rose). Closely spaced peaks (except those within 2 kb of TSS) within a range of 12.5 kb were merged together, followed by the measurement of input and H3K27Ac signals. These merged peaks were ranked by H3K27Ac signal and then classified into SEs or TEs. SEs were assigned to the nearest genes. SE-associated lncRNAs were screened from the nearest genes.

### Bioinformatics analysis

Prognostic information of lncRNAs was obtained from TCGA (The Cancer Genome Atlas) and analyze by GEPIA (http://gepia.cancer-pku.cn/). Expression pattern of LINC01004 in hepatocellular carcinoma was analyzed by using GEO database (GSE67260, GSE55092, GSE58043, GSE62232, and GSE6764). E2F1 ChIP-seq and ATAC-seq data in HepG2 cells were obtained from ENCODE project (ENCSR717ZZW and ENCSR042AWH). ChIP-Seq and ATAC-seq data analysis were performed as described previously. The AQUAS pipeline (https://github.com/kundajelab/chipseq_pipeline) was used to processed ChIP-Seq and ATAC-seq data. Reads was filtered by fastqc (http://www.bioinformatics.babraham.ac.uk/projects/fastqc/) to remove duplicate reads and low quality bases. Then reads were aligned to the reference human genome (hg38). MACS2 (https://pypi.python.org/pypi/MACS2) was used for peak calling.

### Quantitative real-time PCR (qRT-PCR) for gene expression

LncRNAs and mRNAs were reverse transcribed using RevertAid First Strand cDNA Synthesis Kit (Thermo, Wuhan, China) in accordance with the manufacturer’s instructions. The qRT-PCR were performed using a standard UltraSYBR Mixture (CWBIO, Beijing, China) in the Roche LightCycler 480 system (Roche, Mannheim, Germany) according to the manufacturer’s instructions. Human *β-actin* gene was used as endogenous control genes for qRT-PCR. The qRT-PCR data were analyzed using the 2^−∆∆CT^ method, as previously described [22].

### Western blot

Cells were split by radio immunoprecipitation assay (RIPA) buffer (Beyotime, Jiangsu, China) and supplemented with 0.01% of phenylmethanesulfonyl fluoride (PMSF) (Beyotime, Jiangsu, China). The protein was separated by sodium dodecyl sulfate (SDS) polyacrylamide gel electrophoresis, and then was transferred to a polyvinylidene Fluoride (PVDF) membrane (Millipore, Boston, USA). Next, the protein was incubated with the corresponding primary antibody and secondary antibody. Antibodies included Cas9 (1: 1000, Proteintech, Wuhan, China), E2F1 (1: 1000, Proteintech, Wuhan, China), and β-actin (1: 1000, Proteintech, Wuhan, China).

### Cell culture and transfection

The human hepatocellular carcinoma cells (HepG2 and SK-Hep-1 cell lines) were maintained at 37 °C in humidified 5% CO_2_ atmosphere containing 5% CO_2_ in Dulbecco’s modified Eagle medium (Hyclone, Logan, USA) supplemented with 10% fetal bovine serum (FBS) (CLARK, Richmond, USA). Cells were plated and grown until they were reached 70–80% confluent. pCMVHA E2F1 was a gift from Kristian Helin [23] (Addgene plasmid # 24,225, https://www.addgene.org/24225/, RRID:Addgene_24225). pLV hUbC-dCas9 KRAB-T2A-GFP was a gift from Charles Gersbach (Addgene plasmid # 67,620, https://www.addgene.org/67620/, RRID:Addgene_67620) [24]. Then plasmids and siRNAs (GenePharma, Suzhou, China) were transfected using lipofectamine 3000 (Invitrogen, Carlsbad, USA).

si-NC: sence 5′- UUCUCCGAACGUGUCACGUTT-3′,

antisence 5′- ACGUGACACGUUCGGAGAATT-3′;

si-LINC01004-1: sence 5′- GGUUCAAGGUAUAAGCUAAACTT-3′;

antisence 5′- UUAGCUUAUACCUUGAACCUATT-3′;

si-LINC01004-2: sence 5′- GGGAAUAUGUUGUGUUCUAAUTT-3′;

antisence 5′- UAGAACACAACAUAUUCCCGATT-3′;

### Chromatin immunoprecipitation (ChIP)

Chromatin Immunoprecipitation was performed as described previously [25]. Briefly, Cells were initially cross-linked with 1% formaldehyde, and followed by nuclear extraction. Chromatin/DNA complex was sheared in a Bioruptor Sonicator (Diagenode, Denville, NJ). Sonicated lysates were cleared and incubated overnight at 4 °C with magnetic beads coupled with E2F1 antibody (Proteintech, Wuhan, China). Precipitated immune-complex was washed, and DNA was eluted and analyzed by qRT-PCR.

### Luciferase reporter assays

Enhancers were cloned into firefly luciferase reporter vector pGL3-Promoter (Promega, Madison, WI). The indicated cells were transfected using lipofectamine 3000 (Invitrogen, Carlsbad, USA) and A Renilla luciferase plasmid was co-transfected as a normalization control. pGL3-basic plasmid was used as negative control. Luciferase reporter activity was measured using the DualLuciferase Reporter Assay System (Promega).

### Cell counting and colony-formation assay

In cell counting assays, 5*10^4^ cells were seeded onto 12-well plates, and cultured for 3 days. Cell counting assays were performed at 24 h, 48 h, and 72 h after inoculation. In colony-formation assay, 2000 cells were seeded onto 6-well plates, and cultured for 2 weeks. Cells were fixed with methanol and stained with crystal violet.

### Transwell migration assay

Cell migration assay was performed in Boyden Chamber. Briefly, 1*10^5^ cells in fetal bovine serum–free medium were seeded onto the membrane with 8-mm pores of the top chamber (Thermo Fisher Scientific), with the bottom chamber containing regular medium with 20% fetal bovine serum. After 24 h (SK-Hep-1 cell) or 48 h (HepG2 cell), the membranes were washed, fixed, and stained with crystal violet, and migrated cells were quantified.

### Wound-healing and transwell assays

For wound-healing assays, a wound was scratched by a 10 μl pipette tip when the cell layer of SK-Hep-1 or HepG2 cells reached about 90% confluence. Cells were continued cultured at 37 °C with 5% CO2, and the average extent of wound closure was quantified.

### CRSIPR-dCas9

gRNAs were designed by E-CRISPR (http://www.e-crisp.org/E-CRISP/). The gRNA sequences are shown in Additional file 2: Figure S2. Then the gRNAs were inserted into the pLV hU6-sgRNA hUbC-dCas9-KRAB-T2a-GFP vector (addgene, #71,237). The recombinant plasmids were transfected with Lipofectamine 3000 (Thermo Fisher, L3000001).

### Statistics

The difference between two groups was calculated using Student’s t test. One-way ANOVA analysis with Dunnett’s test was used for multiple comparisons. The significance of expression association between different genes was calculated using Spearman’s correlation. A *P* value of less than 0.05 was used as the criterion of statistical significance. All analyses were performed with SPSS software package (Version 16.0, SPSS Inc.) or GraphPad Prism (Version 5, GraphPad Software, Inc.).

## Results

### Identification of super-enhancer-associated lncRNAs in HCC

**Fig. 1:**
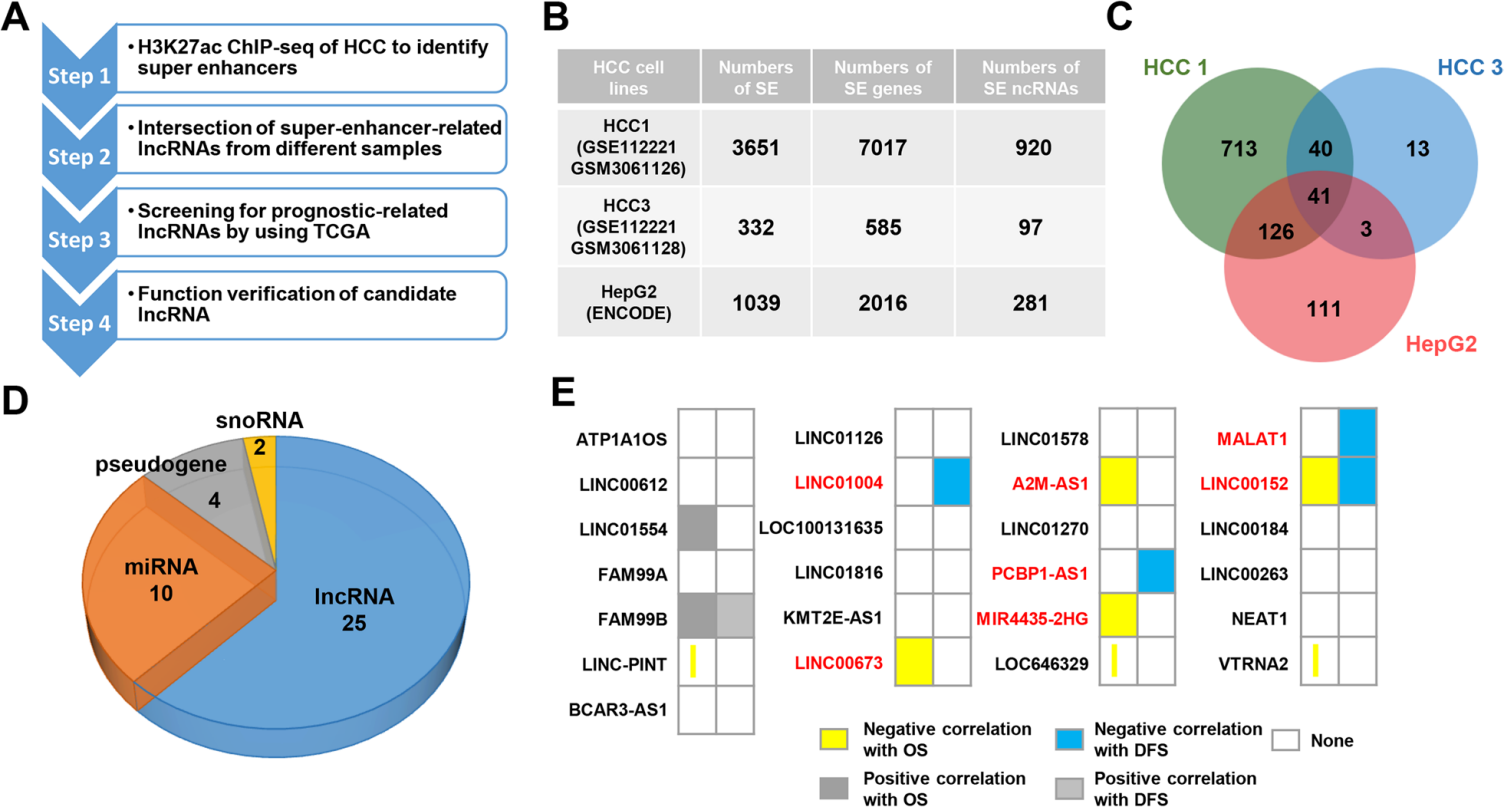
Identification of SE-associated lncRNAs in HCC. **A** Schematic diagram of SE-associated lncRNAs screening. **B** Statistics of SEs and SE-associated genes in HCC. **C** Venn diagram of SE-associated ncRNAs from HCC1, HCC3, and HepG2 groups. **D** Distribution of different types of SE-associated ncRNAs. **E** Correlation between SE-associated lncRNAs and prognosis of HCC patients. OS means overall survival; DFS means disease-free survival

To identify SEs in HCC, H3K27ac ChIP-seq data from HepG2 cell line (ENCODE, ENCSR000AMO) (HepG2 group) and two HCC tissues (GEO, GSE112221) (HCC1 group, GSM3061126 and HCC3 group, GSM3061128) were analyzed (Fig. 1A). As shown in Fig. 1B, we identified 3,651, 332, and 1039 SEs, respectively, in HCC1 tissue, HCC3 tissue, and HepG2 cell line by using ROSE software. The SEs were assigned to the nearest genes. According to the genome annotation, 920, 585, and 2016 SE-associated non-coding RNAs (ncRNAs) were obtained, respectively (Fig. 1B). There were 41 SE-associated ncRNAs were obtained in the intersection of the three groups (Fig. 1C), in which there were 25 lncRNAs (Fig. 1D). Then The Cancer Genome Atlas Program (TCGA) was used to analyze the correlation between these 25 SE-associated lncRNAs and the prognosis of HCC patients. As shown in Fig. 1E, there was 7 lncRNAs (*LINC01004*, *LINC00673*, *MIR4435-2HG*, *A2M-AS1*, *MALAT1*, *PCBP1-AS1*, and *LINC00152)* significantly negatively correlated with HCC patients’ prognosis, in which *LINC00673* [26], *MIR4435-2HG* [27], *MALAT1* [28], *PCBP1-AS1* [29], and *LINC00152* [30] have been reported to promote the progression of hepatocellular carcinoma, demonstrating the important oncogenic function of SE-associated lncRNAs. The roles of *LINC01004* and *A2M-AS1* in cancer were still unknown. In this study, *LINC01004* was choose for the following study.

### Feature identification of *LINC01004*

**Fig. 2:**
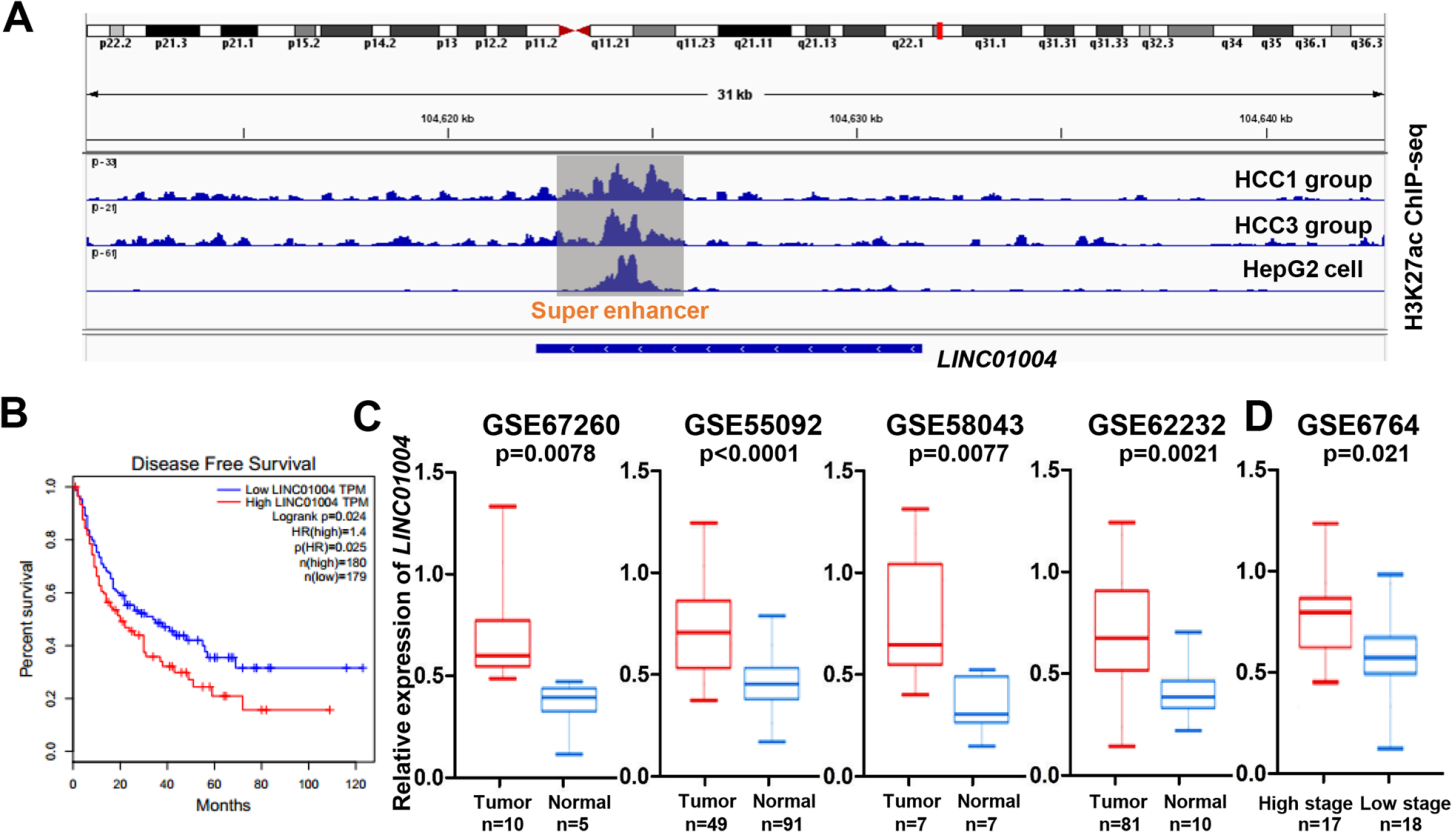
Feature identification of *LINC01004*. **A** The IGV results of H3K27ac ChIP-seq signals from HCC1, HCC3, and HepG2 groups. The red box represents SE. **B** Disease free survival analysis of HCC patients with high *LINC01004* expression and low *LINC01004* expression from TCGA program. **C** The expression pattern of *LINC01004* in HCC tumor and normal tissues in GSE67260, GSE55092, GSE58043, and GSE62232 datasets. **D** The expression pattern of *LINC01004* in HCC tumor tissues with high stage and low stage in GSE6764 dataset

*LINC01004* was a long intergenic non-protein coding RNA on chromosome 7, whose biological function remained to be further studied. Online software CPC (http://cpc.gao-lab.org/) was used to confirm the coding potential of *LINC01004*, and showed that *LINC01004* had no coding potential (Table 1). The IGV results of H3K27ac ChIP-seq signals in Fig. 2A demonstrated there was a SE element within *LINC01004.* Then, we analyzed the H3K4me1(GSM3061121 and GSM3061123), and H3K4me3 (GSM3061131 and GSM3061133) ChIP-seq signals near *LINC01004* SE element, and the results showed that H3K4me1 signal was located in the SE region, while H3K4me3 signal was mainly located in the promoter region (Additional file 1: Figure S1). According to the prognosis data from TCGA Program, HCC patients with high *LINC01004* expression had significantly poorer disease-free survival (DFS) than patients with low *LINC01004* expression (Fig. 2B). Then we analyzed the expression pattern of *LINC01004* in HCC tumor and normal tissues in multiple GEO datasets (GSE67260, GSE55092, GSE58043, and GSE62232). As shown in Fig. 2C, the expression of *LINC01004* in tumor tissues was significantly higher than that in normal tissues. Moreover, we also found that the expression of *LINC01004* in tumor tissues with high stage was also significantly higher than that with low stage in GSE6764 dataset (Fig. 2D). These results suggest that *LINC01004* was a highly expressed SE-associated lncRNA in HCC tumors.

**Table 1:** The predicted coding potential of *LINC01004*

Gene	Coding score	Coding/non-coding
*LINC01004*	− 0.215836	Non-coding
*GAPDH*	12.8603	Coding
*NEAT1*	− 1.21743	Non-coding

Coding score < 0 means no coding potential; Coding score > 0 means certain coding potential

### Inhibition of super-enhancer activity can decrease *LINC01004* expression

**Fig. 3:**
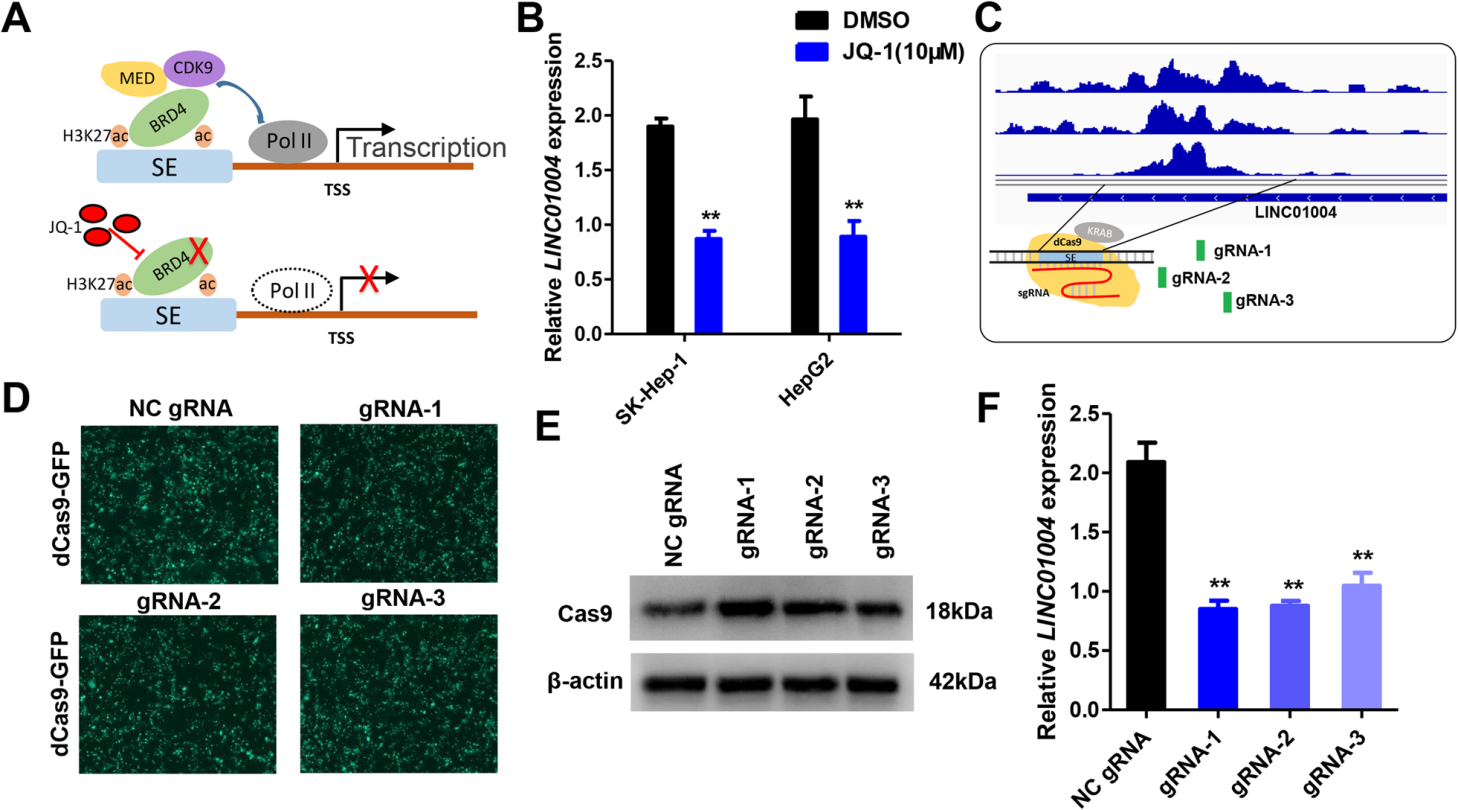
Inhibition of SE activity can decrease *LINC01004* expression. **A** Schematic diagram of JQ-1 inhibiting SE activity. **B** The expression of *LINC01004* in SK-Hep-1 and HepG2 cells after JQ-1(10 μM) treatment for 48 h. **represents *P* < 0.01. **C** Schematic diagram of gRNAs design and CRISPR-dCas9. **D** GFP fluorescence results of HepG2 cells after gRNA-dCas9 vectors transfection for 48 h. **E** Western blot results of HepG2 cells after gRNA-dCas9 vectors transfection for 48 h. **F** The expression of *LINC01004* in HepG2 cells after gRNA-dCas9 vectors transfection for 48 h. **represents *P* < 0.01

BRD4 can interact with hyperacetylated histone regions on chromosome and accumulate on SE elements to promote gene transcription [31]. JQ-1 was a BET-bromodomain inhibitor, which can lead to preferential loss of BRD4 at SEs and consequent transcription elongation defects that preferentially impacted genes with SEs [32] (Fig. 3A). To confirm the regulation of SE on *LINC01004*, hepatoma cell lines SK-Hep-1 and HepG2 were treated with 10 μM JQ-1 for 48 h. As shown in Fig. 3B, the JQ-1 treatment significantly inhibited *LINC01004* expression. Moreover, we designed three gRNAs targeting SE element of *LINC01004* and used CRISPR-dCas9 system to inhibit the SE activity (Fig. 3C and Additional file 2: S2). Fluorescence and Western blot results in Fig. 3D and E showed that dCas9 protein was successfully transfected into the HepG2 cells. qRT-PCR results in Fig. 3F demonstrated that gRNA-1 and gRNA-2 can decrease *LINC01004* expression without effecting nearby genes of *LINC01004* (Additional file 3: Figure S3). The above results indicated that the inhibition of SE activity can decrease *LINC01004* expression.

### E2F1 combined with *LINC01004* super-enhancer to promote its expression

**Fig. 4:**
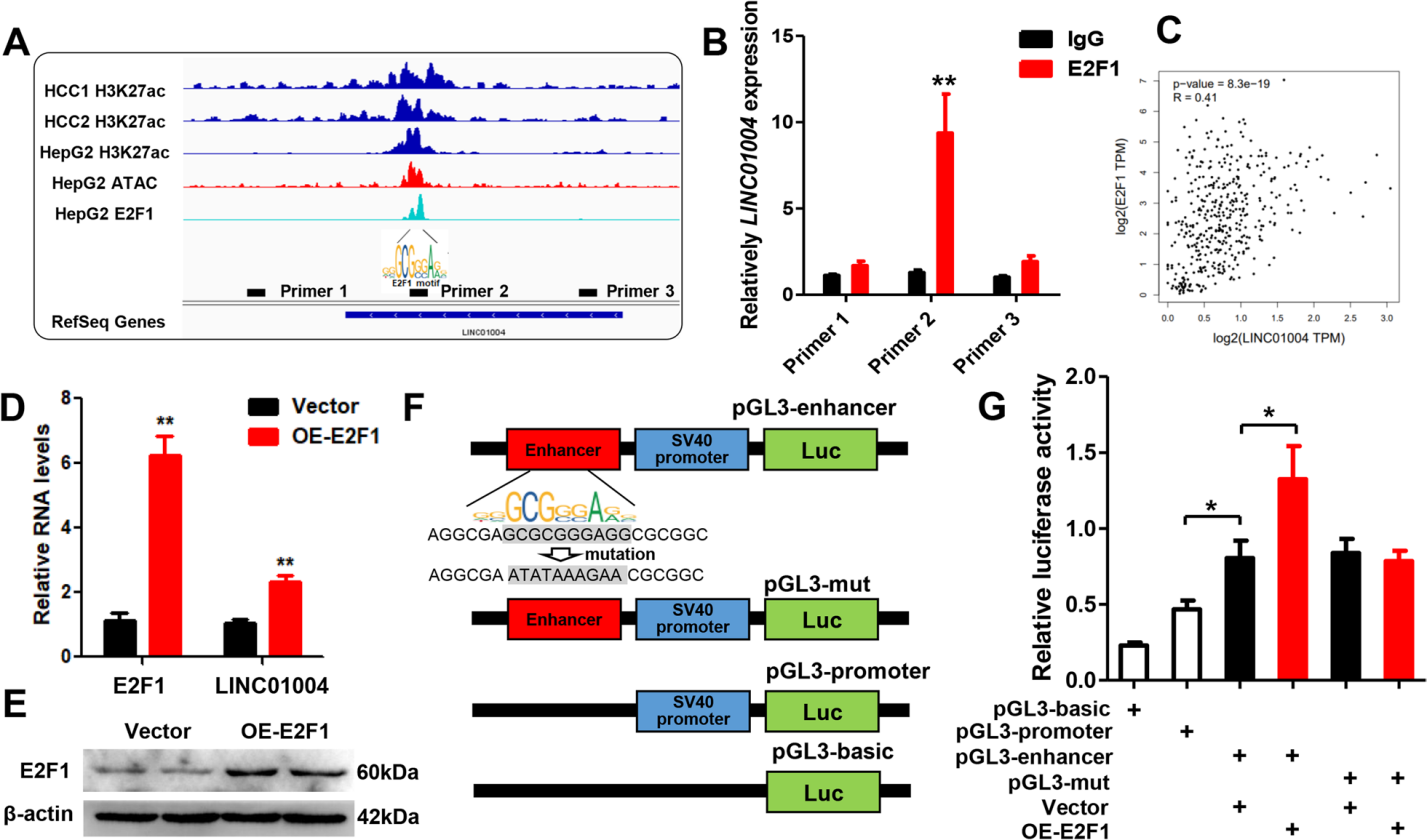
E2F1 combined with *LINC01004* SE to promote its expression. **A** The IGV results of H3K27ac ChIP-seq, E2F1 ChIP-seq, and ATAC-seq. **B** E2F1 ChIP-qPCR results of HepG2 cells. IgG antibody was used as negative control. **C** Correlation analysis of *E2F1* and *LINC01004* in tumor tissues of TCGA HCC patients. **D** qRT-PCR results of *E2F1* and *LINC01004* in HepG2 cells. **E** Western blot results of E2F1. **F** Schematic diagram of pGL3-enhancer vectors. **G** Dual-luciferase reporter system assay of pGL3-enhancer vector. pGL3-basic vector was used as negative control

To identify the transcription factors combined with *LINC01004* SE element, online software JASPAR (https://jaspar.genereg.net/) was performed. As shown in Fig. 4A, there was an E2F1 binding motif in *LINC01004* SE element. Moreover, E2F1 ChIP-seq and ATAC-seq results of HepG2 cells from ENCODE program demonstrated the interaction between E2F1 and *LINC01004* SE element. Our E2F1 ChIP-qPCR results also confirmed that E2F1 combined with *LINC01004* SE (Fig. 4B). E2F1 was significantly overexpressed in HCC tumor tissues and negatively correlated with the prognosis of patients (Additional file 4: Figure S4). The expression of *E2F1* and *LINC01004* in tumor tissues of TCGA HCC patients was significantly positively correlated (Fig. 4C). Moreover, the expression of *E2F1* and *BRD4* was also significantly positively correlated (Additional file 5: Figure S5). These results implied that E2F1 can bind to *LINC01004* SE to promote its expression. To verify our hypothesis, we transfected E2F1 overexpression vector into HepG2 cells and discovered that E2F1 significantly promoted *LINC01004* expression (Fig. 4D and E). Then the *LINC01004* SE element was insert into the pGL3-promoter vector (named as pGL3-enhancer) (Fig. 4F). As shown in Fig. 4G, dual-luciferase reporter system assay results indicated that *LINC01004* SE were active and E2F1 can promote its activity. When the E2F1 binding site in *LINC01004* SE was mutated, E2F1 could not affect the luciferase activity. The above results demonstrated that E2F1 combined with *LINC01004* SE to promote its expression.

### Knockdown of *LINC01004* inhibited HCC cell proliferation and metastasis in vitro

**Fig. 5:**
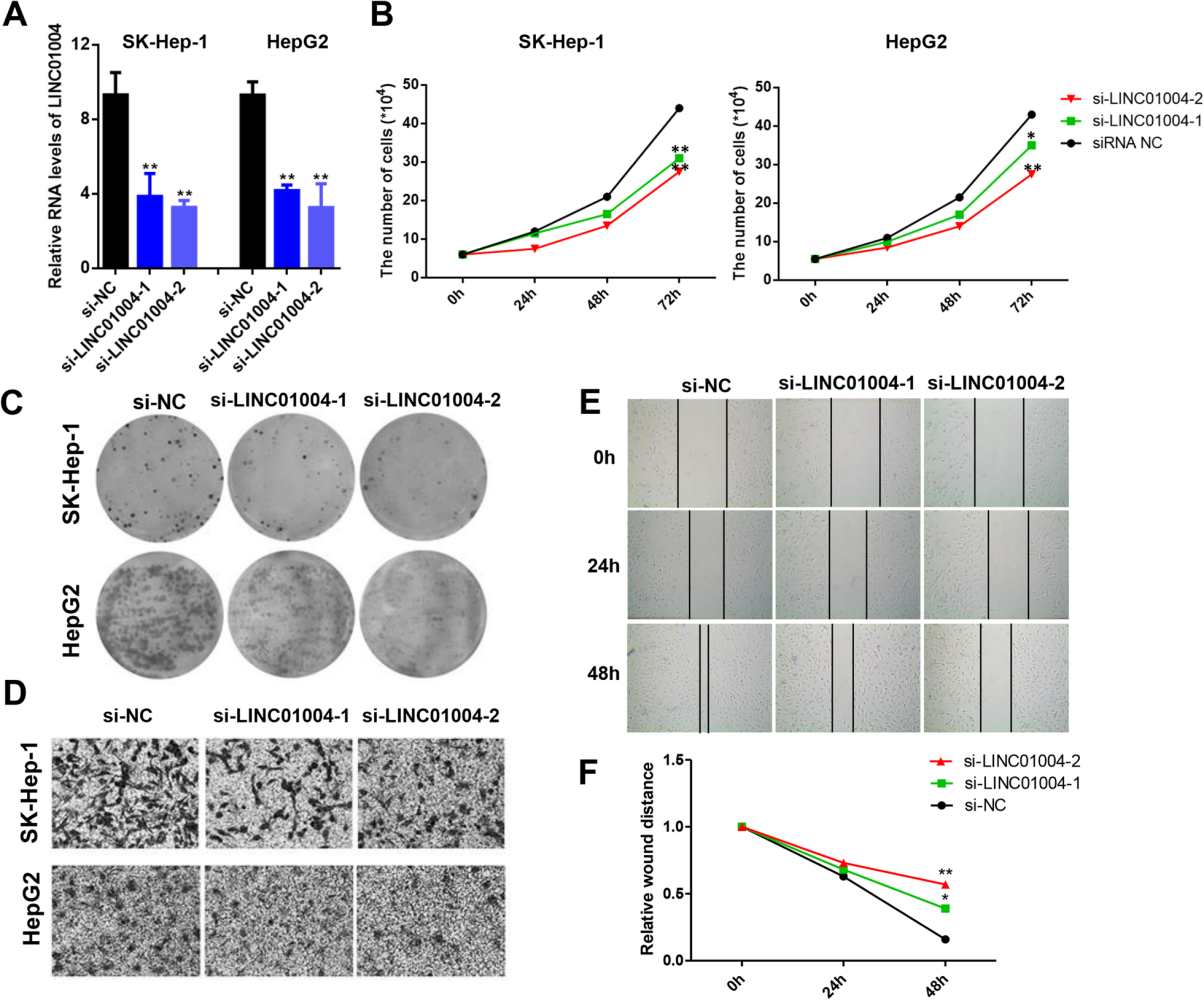
Knockdown of *LINC01004* inhibited HCC cell proliferation and metastasis in vitro. **A** qRT-PCR results of *LINC01004* to detect siRNAs knockdown efficiency. ** represents *P* < 0.01. **B** Cell counting assay of SK-Hep-1 and HepG2 cells after knockdown of *LINC01004*. **C** Colony-formation assay of SK-Hep-1 and HepG2 cells after knockdown of *LINC01004*. **D** Cell transwell assay of SK-Hep-1 and HepG2 cells after knockdown of *LINC01004*. **E** Wound-healing assays of SK-Hep-1 cells after knockdown of *LINC01004*. **F** Quantification result of wound-healing assays. *represents *P* < 0.05. **represents *P* < 0.01

To identify the function of *LINC01004* in HCC, siRNAs was used to decline the expression of *LINC01004*. As shown in Fig. 5A, *LINC01004* expression levels was extremely significantly inhibited after the transfection of siRNAs in HepG2 and SK-Hep-1 cells. Then cell counting assay showed that knockdown of *LINC01004* by siRNAs inhibited HepG2 and SK-Hep-1 cell proliferation (Fig. 5B). Similarly, knockdown of *LINC01004* also inhibited HCC cell colony formation (Fig. 5C). Moreover, cell transwell assay suggested that knockdown of *LINC01004* decreased the metastasis ability of HCC cells (Fig. 5D). The wound-healing assays demonstrated that knockdown of *LINC01089* impaired the motility of SK-Hep-1 cells (Fig. 5E and F). These results indicated that knockdown of *LINC01004* inhibited HCC cell proliferation and metastasis in vitro.

### Knockdown of *LINC01004* inhibited HCC cell proliferation and metastasis in vivo

**Fig. 6:**
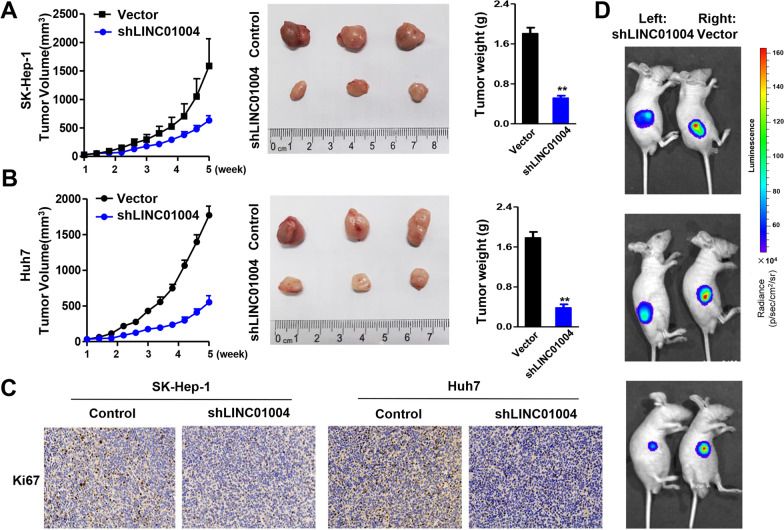
Knockdown of *LINC01004* inhibited HCC cell proliferation and metastasis in vivo. **A** Tumor volume and weight of SK-Hep-1 xenografts in mice. **B** Tumor volume and weight of Huh7 xenografts in mice. **C** Ki67 immunohistochemical results of xenografts. **D** Luciferase activity of intraperitoneal injection mouse model

To identify the function of *LINC01004 *in vivo, generated *LINC01004*-knockdown stable HCC cell lines (SK-Hep-1 and Huh7). We then evaluated the oncogenicity capability of *LINC01004 *in vivo. We found that the growth of the *LINC01004*-knockdown SK-Hep-1 or Huh7 xenografts in mice was significantly retarded compared with that of control xenografts after 5 weeks (Fig. 6A and B). Evidently increased tumor weights were also observed in the *LINC01004*-knockout group compared to the control group (Fig. 6A and B). Ki67 protein levels in xenografts of the *LINC01004*-knockdown group were lower than those in control xenografts (Fig. 6C). Moreover, we used intraperitoneal injection mouse model to confirm the roles of *LINC01004* in cell metastasis in vivo*.* As shown in Fig. 6D, knockdown of *LINC01004* can remarkably suppress liver metastasis of HCC cells in vivo*.* These results indicated that knockdown of *LINC01004* inhibited HCC cell proliferation and metastasis in vitro*.*

## Discussion

Super-enhancers, as active regulatory elements, are capable of driving much higher levels of transcription, and exhibit much stronger lineage- and tissue-specificity compared with typical enhancers [33]. The activity of SEs is related to histone modification, and it is generally considered that the active SE is marked by H3K27ac and H3K4me1, accompanied by lack of H3K4me3 [8]. The ChIP-seq technique is usually used for epigenetics research, and has also been used to analyze genome-wide histone modifications. In the present study, we used H3K27ac ChIP-seq data from HepG2 cell line and HCC tissues to identify SEs in HCC. Moreover, we obtained 25 SE-associated lncRNAs, of which 7 lncRNAs were significantly negatively correlated with HCC patient prognosis. *LINC00673* [26], *MIR4435-2HG* [27], *MALAT1* [28], *PCBP1-AS1* [29], and *LINC00152* [30] have been reported to promote the progression of hepatocellular carcinoma, demonstrating the important oncogenic function of SE-associated lncRNAs.

Particularly, we identify an uncharacterized SE-associated lncRNA, *LINC01004*, as a novel oncogenic factor promoting HCC cells proliferation and metastasis in vitro and in vivo. *LINC01004* was significantly negatively correlated with HCC patient DFS, and the expression of *LINC01004* was upregulated in HCC tumor tissue. However, the molecular mechanism of its oncogenic function were still unclear, which need to be further studied. The abnormally high expression of *LINC01004* in tumor tissue may be driven by SE. BET inhibition including the use of specific chemical BET inhibitors like JQ-1 can significantly inhibit expression of SE-driven genes [34], including *LINC01004* expression. CRISPR-dCas9 system was also a potential way to inhibit SE activity [35]. These results will provide new ideas for the treatment of HCC driven by SEs.

There was exceptionally high degree of enrichment for the binding of transcriptional factors and coactivators on SEs [36, 37]. In this study, E2F1 was found to be a transcriptional factor combined with *LINC01004* SE, and promote *LINC01004* expression. E2F1, a member of E2F family, play important roles in the regulation of many cellular processes including cell proliferation and apoptosis in HCC [38]. E2F1 is highly expressed in HCC to promote HCC progression through a variety of pathways [39–41]. The high expression of E2F1 is one of the important reasons for the high expression of LINC01004.

In conclusion, this study investigated novel super-enhancer-associated lncRNAs and revealed the oncogenic role of a previously unknown lncRNA in liver cancer. *LINC01004* was a liver-enriched lncRNA and it was frequently upregulated in liver cancer tissues. Subsequent functional studies demonstrated that knockdown of *LINC01004* significantly inhibited tumor cell proliferation and metastasis. E2F1 combined with SEs to promote *LINC01004* expression. Inhibition of SE activity could significantly decrease *LINC01004* expression. This finding might provide mechanistic insights into the molecular mechanisms underlying hepatocarcinogenesis and the biological function of SE, and *LINC01004* can serve as a potential therapeutic target for HCC patient.

## Supplementary Information

**Additional file 1**. **Figure S1**: H3K4me1 and H3K4me3 ChIP-seq signals in HCC1 and HCC3 (GSE112221).

**Additional file 2**. **Figure S2**: gRNAs design by E-CRISPR. (A) The sequence and SAE-Score of gRNAs. (B) The location of gRNA targets on genome.

**Additional file 3. Figure S3**: Expression levels of KMT2E and LOC101927902 in HepG2 cells after gRNA-dCas9 vectors transfection for 48 hours.

**Additional file 4**. **Figure S4**. Expression pattern and prognostic correlation of E2F1 in HCC. (A) Expression pattern of E2F1 in HCC. Left: data from TCGA; Right: data from TGCA and GTEx. (B) Prognostic correlation of E2F1 in HCC. Left: Overall survival (OS); Right: Disease free survival.

**Additional file 5**. **Figure S5**: Correlation analysis of E2F1 and BRD4 in tumor tissues of TCGA HCC patients.

## Data Availability

Not applicable.
