# Supplementary figures and images for: E2F1 combined with LINC01004 super-enhancer to promote hepatocellular carcinoma cell proliferation and metastasis
Source: Clin Epigenetics. 2023 Jan 31;15:17. doi: 10.1186/s13148-023-01428-6 (PMC9887888; doi:10.1186/s13148-023-01428-6)

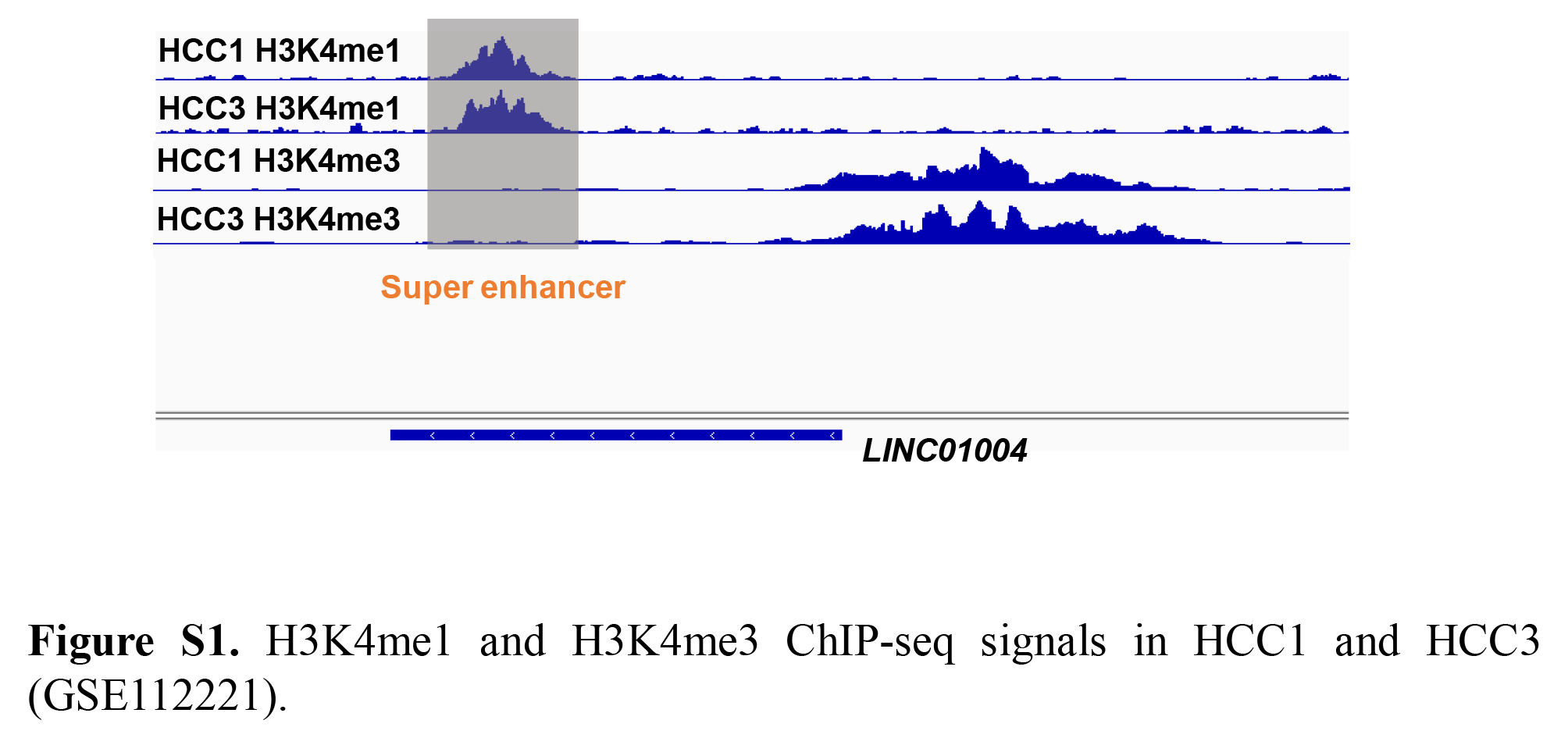

Supplement: Supplementary file 1 — Additional file 1. Figure S1: H3K4me1 and H3K4me3 ChIP-seq signals in HCC1 and HCC3 (GSE112221). [file 13148_2023_1428_MOESM1_ESM.tif]

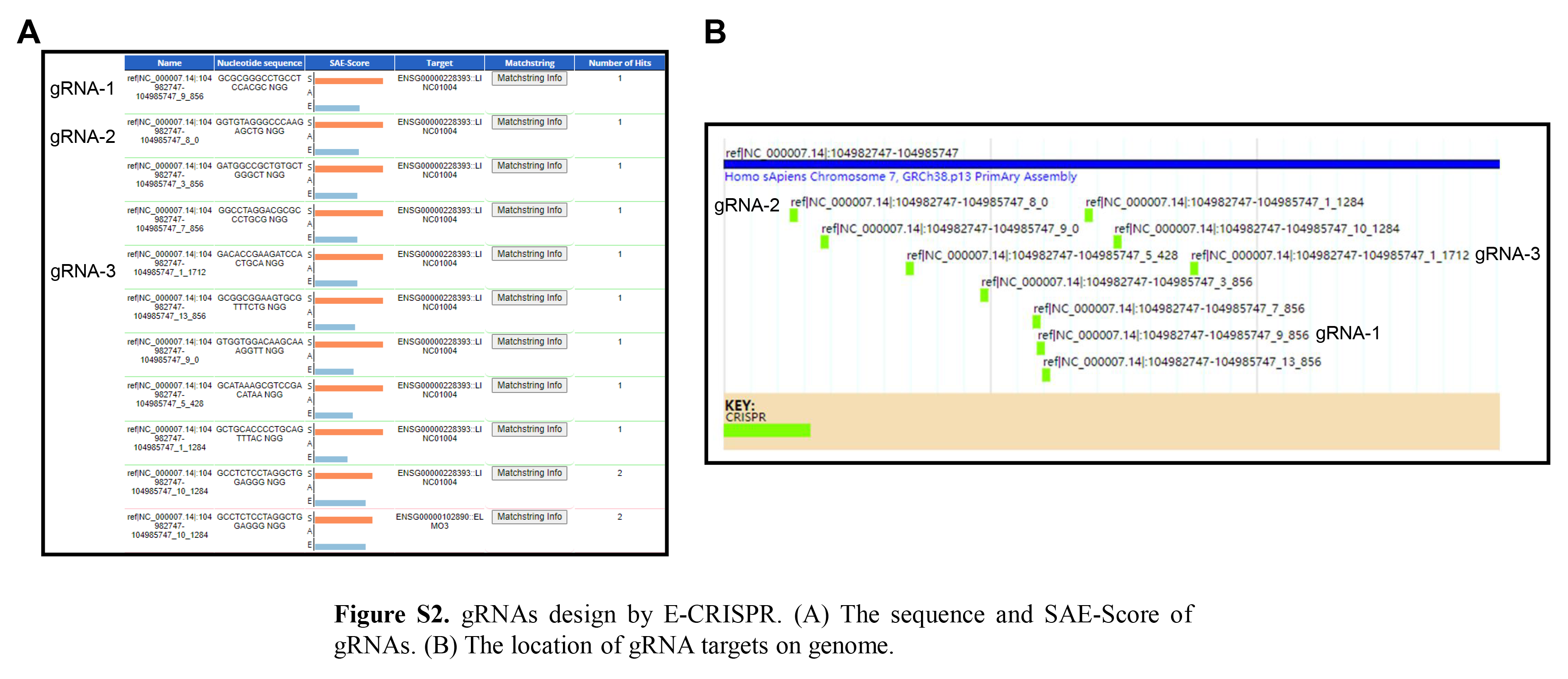

Supplement: Supplementary file 2 — Additional file 2. Figure S2: gRNAs design by E-CRISPR. (A) The sequence and SAE-Score of gRNAs. (B) The location of gRNA targets on genome. [file 13148_2023_1428_MOESM2_ESM.tif]

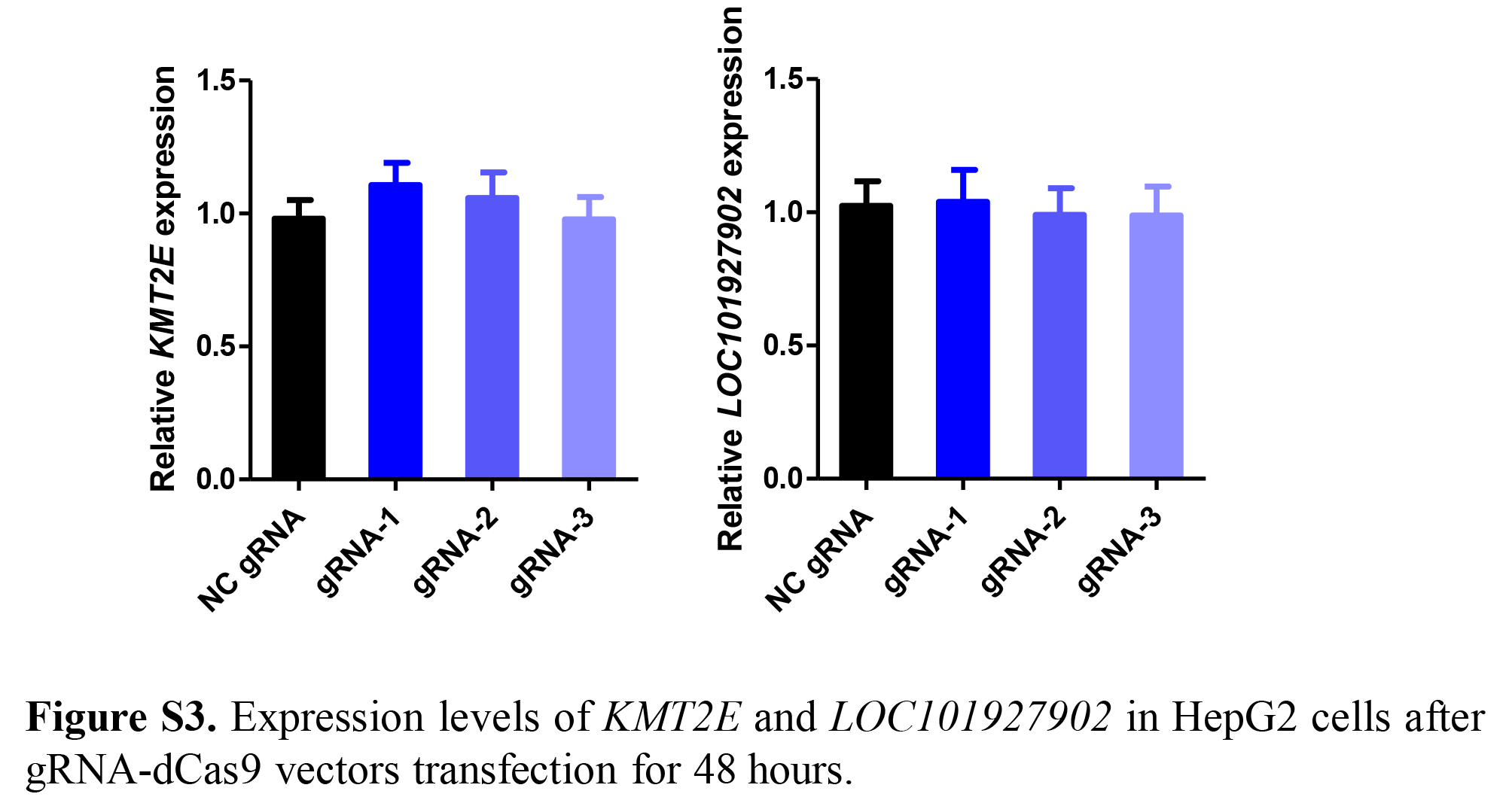

Supplement: Supplementary file 3 — Additional file 3. Figure S3: Expression levels of KMT2E and LOC101927902 in HepG2 cells after gRNA-dCas9 vectors transfection for 48 hours. [file 13148_2023_1428_MOESM3_ESM.tif]

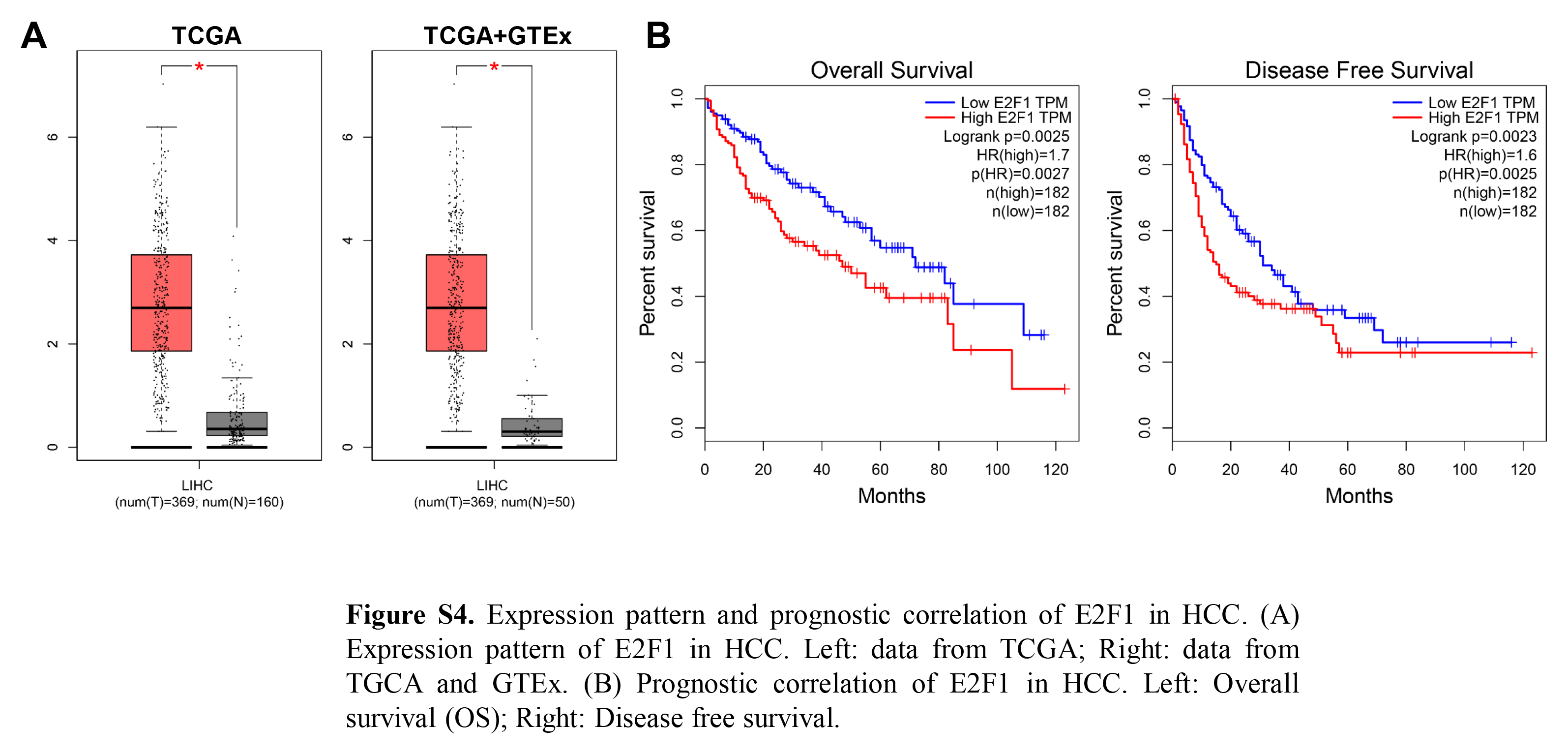

Supplement: Supplementary file 4 — Additional file 4. Figure S4. Expression pattern and prognostic correlation of E2F1 in HCC. (A) Expression pattern of E2F1 in HCC. Left: data from TCGA; Right: data from TGCA and GTEx. (B) Prognostic correlation of E2F1 in HCC. Left: Overall survival (OS); Right: Disease free survival. [file 13148_2023_1428_MOESM4_ESM.tif]

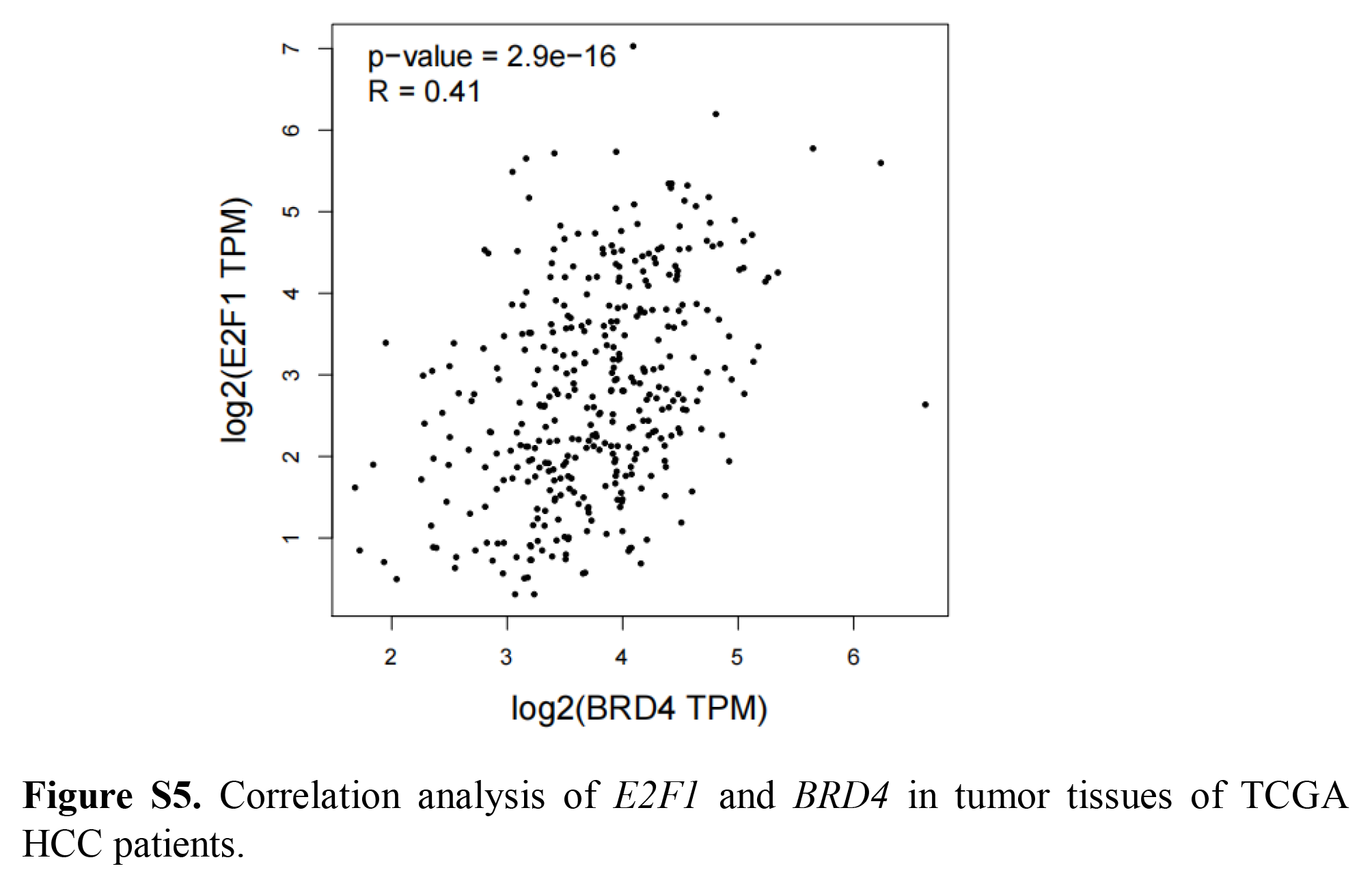

Supplement: Supplementary file 5 — Additional file 5. Figure S5: Correlation analysis of E2F1 and BRD4 in tumor tissues of TCGA HCC patients. [file 13148_2023_1428_MOESM5_ESM.tif]
